# Network‐building by community actors to develop capacities for coproduction of health services following reforms: A case study

**DOI:** 10.1111/hex.13491

**Published:** 2022-04-05

**Authors:** Susan Usher, Jean‐Louis Denis

**Affiliations:** ^1^ Département de gestion, d'évaluation et de politique de santé École nationale d'administration publique Montréal Québec Canada; ^2^ Analyse et management des politiques publiques, École de santé publique Université de Montréal Montréal Québec Canada

**Keywords:** actor–network theory, community engagement, coproduction, health reforms, networks, Québec, social capital

## Abstract

**Introduction:**

Responsive, integrated and sustainable health systems require that communities take an active role in service design and delivery. Much of the current literature focuses on provider‐led initiatives to gain community input, raising concerns about power imbalances inherent in invited forms of participation. This paper provides an alternate view, exploring how, in a period following reforms, community actors forge network alliances to (re)gain legitimacy and capacities to coproduce health services with system providers.

**Methods:**

A longitudinal case study traced the network‐building efforts over 3 years of a working group formed by citizens and community actors working with seniors, minorities, recent immigrants, youth and people with disabilities. The group came together over concerns about reforms that impacted access to health services and the ability of community groups to mediate access for vulnerable community residents. Data were collected from observation of the group's meetings and activities, documents circulated within and by the group, and semi‐directed interviews. The first stage of analysis used social network mapping to reveal the network development achieved by the working group; a second traced network maturation, based on actor–network theory.

**Results:**

Network mapping revealed how the working group mobilized existing links and created new links with health system actors to explore access issues. Problematization appeared as an especially important stage in network development in the context of reforms that disrupted existing collaborative relationships and introduced new structures and processes.

**Conclusion:**

Network‐building strategies enable community actors to enhance their capacity for coproduction. A key contribution lies in the creation of ‘organizational infrastructure’.

**Patient or Public Contribution:**

The lead researcher was embedded over 3 years in the activities of the community groups and community residents. Several group members provided comments on an initial draft of this paper. To preserve the anonymity of the group, their names do not appear in the acknowledgements section.

## 1 INTRODUCTION

Collaborative approaches to public services have been associated with benefits including increased accountability, greater civic engagement, consistent downstream implementation and higher levels of process and programme success.^1^, ^2^, ^3^, ^4^ In health systems, the capacities of people and communities to coproduce outcomes alongside formal providers now appear key to assuring the sustainability, equity and integration of care.^5^

Coproduction is based on interdependence between the capacities different parties bring to solving an issue, and conditions that enable those capacities to be recognized and made.^6^ However, coproduction arrangements are not easily achieved nor maintained over time. Imbalances can result when stakeholders lack the ‘organizational infrastructure to be represented in collaborative governance processes’,^7^ ^(p.551)^ or face barriers to participation. A recent realist synthesis of strategies to engage communities in health service decisions identifies power imbalances as a significant constraint.^8^ ^(p.15)^ The author asks: ‘why (do) professionals and organizations implement community engagement interventions, but then “maintain their business as usual” approach?’ The question reflects a preponderant focus, in contemporary scholarship, on engagement efforts initiated by provider organizations that retain control over the question at hand, the terms of engagement, and actions taken in response. There is scarce evidence that such initiatives have produced a meaningful change in health systems.^9^ Reviews of the public and patient engagement literature^10^ suggest that initiatives both within and outside the provider sphere, as well as links between the two, are needed to create the collaborative dynamics required for coproduction.

This paper is interested in the strategies adopted by service users and their communities to gain the power to influence conditions for coproduction of health services with the public sector. It looks to actor–network theory (ANT) to guide an empirical study of the process of creating these conditions. A longitudinal case study undertaken in an urban neighbourhood in Québec (Canada) explores network‐building efforts by an ad hoc working group (WG) of concerned citizens and community group actors working with seniors, minorities, new immigrants, youth and people with disabilities. The WG formed through concerns that system reforms were compromising access to health services as well as their own ability to help vulnerable people overcome access barriers. The case study aims to understand how community actors use network strategies to establish (or re‐establish) their role as coproducers of health services with the public sector.

The paper begins with an exploration of power and the role of network relations in enabling less dominant actor groups to achieve influence in a field. It then presents the context for the case study, looking at the ‘prehistory’ of collaboration between the public sector and community actors in Québec, along with key elements of recent reforms: these provide the starting conditions and motivation for the efforts of the WG. The study is based on observation of WG meetings and activities over 3 years, interviews with WG members and reviews of WG internal and external communications, which are analysed to trace the evolution of network relations. Findings support network building as a means of developing community capacity, identify venues important to capacity devlopment and suggest health system factors that impede or facilitate collaborative dynamics with communities. These insights point to ways in which coproduction might be supported in both public sector and community spheres.

### 1.1 Collaborative dynamics, power and networks

The collaborative dynamics of coproduction are influenced by power differentials that affect the development, recognition and integration of user and community capacities in public services. In their collaborative governance model, Emerson and Nabatchi^11^ stress recurring social interactions as opportunities to highlight and recognize interdependence, facilitating the assumption by community‐based actors of a problem‐solving role ‘actively engaged in creating what is valued by the public’.^12^ ^(p.446)^ Sociologist Pierre Bourdieu sees power accruing from social capital, defined as ‘the sum of actual or virtual resources that accrues to an individual or group by virtue of possessing a durable network of more or less institutionalized relationships of mutual acquaintance and recognition’ (our translation).^13^ ^(p.2)^ Social capital provides a means of understanding how power can shift among actors with unequal resources to enable the development of mutual understanding and collaboration. The study of network development through the lens of ANT^14^ offers a means of exploring how community actors accumulate social capital to create conditions for the coproduction of health services.

Networks are seen as ‘relatively stable and continuous relationships between institutions, individuals, and/or groups that mobilise resources and information to achieve a collective goal’.^13^ ^(p.2)^ ANT^14^ provides a means of achieving a fine‐grained description of the process of building capacity for social action through networks, where actors ‘converge on common problematizations, negotiate shared interests, engage in new roles and mobilise a critical mass of actors for collective projects’ to build new solutions.^15^ ^(p.166)^ In ANT, the process of linking entities into these ‘sociotechnical networks’ is referred to as ‘translation’. Callon and Ferrary^14^ describe four stages in translation, starting with *Problematization* where relevant entities are identified and connected, and problems and potential solutions are discussed. *Interessement* (generating actor interest) describes negotiation within the network and integration of new entities, and leads to *Enrolment*, or assumption and alignment of roles within the network. Finally, *Mobilization* is the capacity to act that develops in the network.

**Figure 1:**
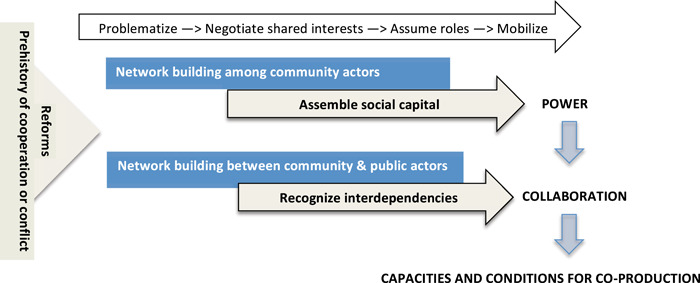
Conceptual model: Network‐building to enable coproduction. Figure 1 presents the conceptual model in this study, based on theories about how less dominant actors gain the power to be recognized as collaborators with more dominant actors in a field. Network‐building among community actors assembles social capital that increases legitimacy to represent an issue and gain the attention of public actors. Network‐building with public actors enables interdependencies to be recognized, making collaboration more likely and enabling new capacities and better conditions for coproduction. The arrow at the top of the figure describes the process of network maturation according to actor–network theory,^14^ which passes through four stages, from problematization to mobilization. The triangle on the left side of the figure depicts the starting conditions for network‐building efforts: both the prehistory of collaborative relations and, in our case, the immediate system reform context

This study is designed around the conceptual model presented in Figure 1. ANT serves as an approach for studying the process of network development *among* community‐based actors to assemble power through social capital, and *between* community‐based and public sector actors to motivate collaboration^7^ through recognition of interdependencies. Network‐building therefore acts on the capacities for coproduction, and on the conditions that enable those capacities to be recognized and brought together to solve problems. The model in Figure 1 also includes the prehistory of relationships between the community and public sectors, and features of recent reforms. These represent starting conditions for the WG's network‐building activities and are essential to understanding how interdependencies are perceived at the outset.^16^ ^(p.718)^

### 1.2 Study context

This section describes the ‘prehistory’ of collaboration between public and community actors in the Québec context, and reforms that led to the starting point of the community‐driven efforts examined in this case study.

Québec has a long legacy of community participation in health and social services. Local community involvement and citizen participation figured prominently in the early design of the province's health and care system.^17^ At the foundation of Medicare in the 1970s, Québec adopted a broad vision of health, combining health and social services in a single ministry and implementing an initial model of primary care that embedded public actors within communities and saw community organizations as partners to meet local needs. Local community health centres (CLSCs) had a mission that included preventive and curative care, social services and community action^18^ with activities strongly oriented to community‐identified needs.^19^ As well, Touzard^20^ and White^21^ point to the tradition of ‘concertation’ in Québec as a consensus‐building strategy that, without assigning ‘formal roles or responsibilities for policy development’, brings community, public and other actors together to ‘tackle shared objectives’.^21^ ^(p.4)^ White describes the ‘tables de concertation’ as governance networks that provide long‐term opportunity to influence policy. However, scholars also note longstanding tensions in Québec between views of community resources as autonomous and arising in a given geographic space or around a given issue, and the perspective that they form a continuum with public services to meet population needs through local and volunteer efforts.^22^ This is pertinent to the idea of coproduction as, rather than weaving together distinct contributions, the ‘complémentariste’ view enlists community‐based actors in a mandated policy programme.

Reforms beginning in 2003 integrated CLSCs into larger Health and Social Service Centres (CSSS), and mission‐based funding of community organizations was replaced by project funding to help with specific programme mandates determined by provincial decision‐makers.^23^ In this context, public sector community organizers felt ‘instrumentalized’^24^ as they were expected to support community mobilization to achieve targets specified by public authorities and not by communities. Some authors also note a decrease in the policy influence of community actors through the ‘tables de concertation’, considering that these venues eventually increased the layers separating public services and community actors and made upward communication of community concerns more difficult.^25^ Most recently, major reforms introduced in 2014–2015 centralized governance in the health system, consolidated 182 provider establishments into 32 vertically Integrated Health and Social Service Centres (CI[U]SSS) for the province's 8.4 million inhabitants (Bill 10), heavily promoted physician‐led Family Medicine Groups (FMGs) as the privileged model of primary care (including transferring nurses and other health professionals from public establishments into the FMGs), and established centralized access portals in each CI(U)SSS for health and care services. These structural reforms reduced statutory opportunities for public and local community participation.^26^, ^27^, ^28^, ^29^

These features of the Québec system represent the prehistory of relations and reform context depicted in Figure 1 as driving and influencing the efforts of community‐based actors in the present case study. The vocabulary of ‘concertation’ and community action persists, but recognition of the distinct contribution of community actors is compromised, and successive waves of reform have eroded opportunities for community and user participation in decision‐making. The immediate reform context provides a privileged opportunity to explore coproduction dynamics: by disrupting established orders, they generate response and thereby help reveal factors that enable and impede community engagement and collaboration between community and public sector actors.

## 2 METHODS

### 2.1 Case selection

In the years following the implementation of the latest reforms in Québec, a number of community‐based initiatives emerged to understand the reconfigured system and difficulties experienced by community residents in accessing health and care services. The WG was selected for this case study as an early initiative involving community groups working with vulnerable populations along with community residents. The WG provided an opportunity to look at how a range of access issues was perceived by actors who had recognized bridging roles between service users and public providers, as well as busers themselves. The group also appeared committed to a continuing effort, while other initiatives were more punctual, with several focusing on mobilizing community residents around a particular change to a local service or facility. The lead researcher learned about the WG through follow‐up on a community meeting she attended to find out about services at a CLSC, contacted a group member, and was invited to attend an early meeting. She presented her doctoral research programme on public and patient engagement and the transformation of health systems and her wish to study the WG's progress to understand how public engagement develops and contributes to transformation. The WG members agreed to have the researcher study their initiative over an open‐ended period of time. The study protocol received ethics approval from the lead author's academic institution, and WG members reviewed and signed written informed consent for the researcher to observe meetings, consult materials shared within the group and conduct individual interviews.

### 2.2 Case description

Table 1 presents the characteristics of eight community actors in an urban neighbourhood who formed the ad‐hoc WG in late 2016. The table details the roles they play within the community and the types of activity they undertake to assure that all community residents can obtain the services they need. They were concerned about the opacity of reforms, and the loss of legitimacy and connections with health system actors they needed to play their roles effectively. They held monthly meetings beginning in January 2017. The group's statement of purpose, agreed in June 2017, reads:

*The Working Group on health care is a collaborative effort between residents and community organizations to evaluate the level of need for and access to healthcare services among people living in our neighbourhood. Our mission is to help ensure that everyone living here has the information and resources necessary to access healthcare services, to promote the health and well‐being of the neighbourhood, and to seek opportunities for collaboration which lead to improved access for all, with a particular focus on isolated and vulnerable residents*.

**Table 1:** Characteristics of original working group (WG) members

Member characteristics	Sources of knowledge
*WG member 1*	Local stakeholder meetings to identify community needs
Community organization, territory‐based to house, coordinate and support community groups and undertake initiatives concerning whole territory population	Participates in several ‘tables de concertation’
	Regular contact with public sector community organizers
	Events and consultations organized with community residents, including strategic planning exercise pointing to a need for community action to address changes arising from 2015 health system reforms and improve access, capacity, diversity and quality of public and community services
*WG member 2*	Experience of working to obtain CLSC in 1970s + former elected CLSC board member
Local resident, retired health professional	Member and former member/board member of various local organizations and ‘tables de concertation’
*WG member 3*	Provision of direct services to minority residents
Community group: Advocacy and services for minority residents	Hears people's need for cultural safety and trust when seeking help
	Helps people with problems arising from discomfort in communicating with public service providers
	Member of various ‘tables de concertation’ and diversity committees of public organizations
	Assembles experts from other community and public organizations to try and develop solutions
*WG member 4*	Participates in ‘tables de concertation’, provincial networks, community organizations
Community group: Advocacy and services for seniors	Focus on the quality of life of low‐income seniors
	Provides direct services for seniors
	Produced age‐friendly cities survey
	Collaborates with public sector providers to design and obtain funding for projects
	Works to ensure public sector actors are aware of community services (i.e., transport to medical appointments) and provide their patients that information
*WG member 5*	Outreach in the community to identify citizens who may need information, help, referrals
Community group: Outreach and support for vulnerable seniors	Intervenes with vulnerable seniors
	Links people with community and institutional resources
	Supports people to access services
*WG member 6*	Provides direct support to new immigrants
	Coordinates ‘table de concertation’
Community group: Supports for new arrivals	Maintains a listing of resources for new arrivals, including refugees
	Fills requests for translation/interpretation services for public sector health organizations
*WG member 7*	Issues raised within public, community and national organizations and vast personal network of contacts in the health and social services sector and community sector
Local resident, prominent figure in local community development, past and present board member/chair of local and national organizations	Personal experience as user and caregiver
*WG member 8*	Issues raised in various community groups and within the healthcare organization
Local residents, retired nurse, member several community groups, volunteer at healthcare establishment	Personal experience of service gaps

Between January 2017 and December 2019 (note: This represents the study period: The WG continued its activities into 2020, though faced disruptions after the Covid‐19 pandemic), WG members met monthly at the offices of one of the community groups involved. Members working in community organizations participated as part of their work; community members participated as volunteers. Meeting agendas and minutes were prepared and circulated, with members assuming various research and outreach tasks between meetings. Some 20 invited guests from healthcare establishments participated in WG meetings over the first 3 years. Members who attended external meetings or events reported relevant information back to the WG. Two additional members joined in the second year: one from a community organization for people with disabilities, one a retired social worker and community resident.

This study employs qualitative methods to capture the rich detail of the network‐building processes^30^ undertaken by the WG. Data were collected through observation, document review and interviews between January 2017 and December 2019. Observation notes were kept on monthly meetings of the WG, of member participation in external activities, and of activities organized by the WG. Observation focused on contributions during meetings, interactions among WG members and with guests; at external events and activities, observation was interested in interactions between WG members and other actors, the types of issues discussed, opportunities for input and feedback, and interest generated and expressed in WG initiatives. Documents included meeting minutes along with material introduced and shared among members, email communications and material distributed by the WG to the broader community and to public sector actors. Documents helped distinguish between what was discussed internally and how those discussions solidified into more concrete actions or proposals. Interviews were conducted with the eight original members of the WG at staggered time points during 2018 according to participant availability. These focused on members' sources of knowledge about access issues, relationships with other actors related to health and care services (pre‐existing and formed through the WG), concerns that prompted them to join the WG, and ways in which the WG helped them address these concerns. Interviews were recorded with participant consent, transcribed verbatim and anonymized. Observation notes, interviews and documents were combined in a single database to triangulate and complement one another.^31^ The data that support the findings of this study are available from the corresponding author upon reasonable request.

### 2.3 Analysis

The first stage of analysis maps the network‐building efforts accomplished through the WG. Network analysis provides an empirical entry point to study the dynamics of network formation.^32^ Interviews with WG members, meeting minutes and observation notes, agendas and email correspondence of group members were mined to trace: (1) the network ties related to health and care services each actor had coming into the WG, and (2) the WG's exploitation of these ties and formation of new links over time. All actor‐groups mentioned in interviews, meeting minutes and communications were entered into a database and were coded according to their relationship to WG members and activities. This coding was processed using social network analysis software (Gephi)^33^ to trace the phenomenon of network development: pre‐existing ties of WG members, connections introduced into WG deliberations (i.e., as guests or resources), and pre‐existing and new connections made by the WG. Network mapping helps explore the capacity to develop relationships,^34^ highlighting bridging actors and events that enable the network to come together and expand.^35^ While often used in quantitative analysis, authors have highlighted its value in qualitative research^36^ to trace the relationships developed in a given initiative.

Second, this evolution is explored through the lens of ANT^14^ to better understand how network building develops capacities for coproduction. ANT posits that it is the connections between various entities (social actors, ideas, resources, etc.) that produce an effect on social action.^37^ As proposed by Callon and Ferrary,^14^ we analyse data to document the chain of events that produce new connections and the preliminary effects these have on capacities and conditions for coproduction. Given that harder outcomes (i.e., better access to health and care services) would emerge only over the long term, the object of observation is the structure and dynamic of relationships.^23^

## 3 RESULTS

### 3.1 Network building

**Figure 2:**
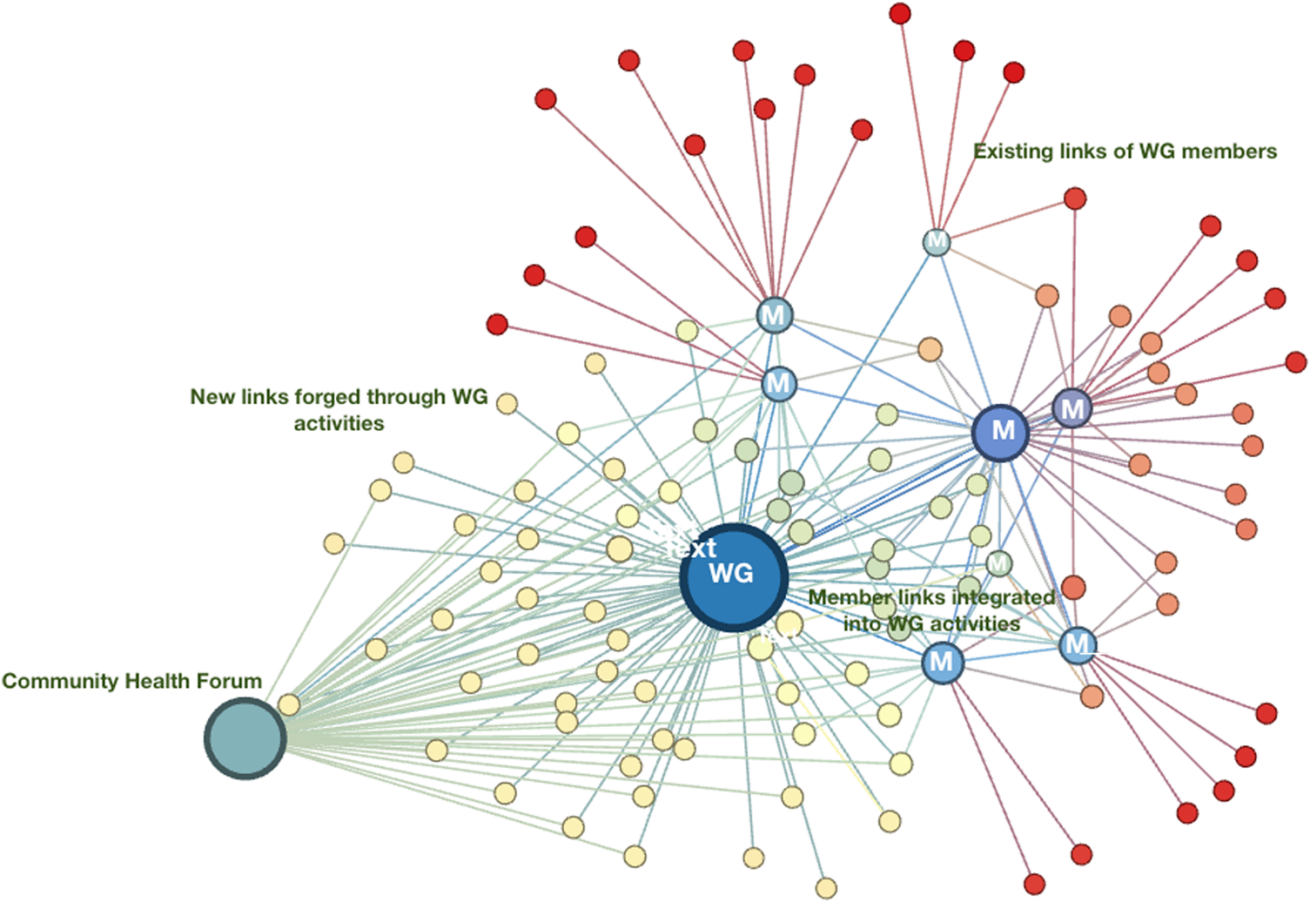
Network development accomplished by the working group: 2017–2019. Figure 2 graphically depicts a point‐in‐time view of the network building accomplished by the working group over its first 2 years. The network mapping is egocentric, meaning it only considers links from the perspective of the working group: the pre‐existing contacts of members related to health and care services, and additional links formed through the group's activities. The mapping does not depict relationships between other network actors, though these surely exist. The large blue node in the centre of the figure is the working group. The blue nodes marked ‘M’ are the eight original members of the group, with lines extending to the links they had at the outset that were relevant to health and care services. Some of these links (the red dots) fed into the knowledge and perspectives members brought into the group, while others (the yellow dots) were more directly exploited in working group activities, brought in as ‘guests’ to working group meetings or enlisted to participate in events. The large green node on the left represents a Community Health Forum organized by the WG in its second year of activity.

Results are based on analysis of data from interviews with the eight original members of the WG presented in Table 1, observation notes from 72 h of WG meetings and over 40 h of WG member participation in events, and over 200 documents, including meeting minutes, internal WG communications and emails between WG members and other actors. Figure 2 presents a mapping of the network building achieved by the WG over the study period. It illustrates that the creation of the WG brought together community actors with knowledge and insights drawn from the constituencies they worked with, but also from pre‐existing links with other community organizations, politicians, researchers, health system providers and managers. Deliberations within the WG (represented by the large blue node in the centre) were therefore, at the outset, informed by this aggregated set of some 70 contacts related to health and care services. Over the study period, a number of these contacts attended WG meetings as guests to explore particular questions: family physicians working in FMGs, front‐line professionals, managers and communications officers in the CI(U)SSS, researchers, and so forth. We further see, in the multiple nodes gravitating around and to the left of the central blue node, that the WG forged a new set of links to explore diverse perspectives on access issues and potential solutions. The organization of a Community Health Forum (the green node at the far left) enabled the WG to bring together system actors, community actors and neighbourhood residents.

What Figure 2 describes is the machine behind the creation of social capital. We now look at how this web was put in action to enable community actors to better understand the postreform system and effectively act within it to address access issues.

### 3.2 Actor‐network development

ANT considers that power for action accrues as networks mature and develop consensus around paths to action, passing through stages of problematization, development of actor interest, role assumption and broader mobilization towards a consensus mission. This next section explores the work undertaken by the WG along these stages to influence conditions for coproduction within the health services field.

#### 3.2.1 Problematizing access issues

Table 1 describes the experience and knowledge WG members brought together. In early meetings, they pooled their perspectives to assemble a clearer vision of access difficulties: people's increasing distress and level of deterioration, rigid intake protocols, gaps in postdischarge care, difficulties with coordination and access to primary care, long waits, public programme cuts, and so forth. Front‐line health services previously available at the local CLSC had been moved outside the neighbourhood, a decision that disregarded recommendations from an earlier community consultation in which several WG members had been involved.

*And we didn't know what was happening in the building that was supposed to be ours. The Agency comes and talks to the community to develop a strategic plan. But then they don't necessarily share their plan, and we might not recognize our input once it's in place. The community input didn't really have any impact, because then the decision was made [for the service governance unit] to become even bigger*. WG member 2

As well, front‐line personnel employed by the CI(U)SSS were being moved from CLSCs to FMGs, which are run by physicians and disconnected from local communities.

*A lot of front‐line services were moving, and still are, from CLSCs into FMGs, with a whole range of implications for service users, many of which we don't fully understand. These (FMGs) are actors who are not as tied into our community network, and yet they're some of the most important players on our territory and their influence seems to be increasing*. (WG member 1)

Community actors in the WG found themselves less able to support vulnerable community members. As one WG member said, access to public services depends on ‘how comfortable the community you serve is in coming to you for that help’ (WG member 4) and community groups often play a bridging role. WG members found that longstanding relationships with individual public sector nurses, social workers and community organizers with whom they could troubleshoot access problems were increasingly fragile; these professionals were being moved around, faced new work demands and were less available. This also disrupted connections needed to ensure public sector referrals to community resources: ‘It takes someone on the inside to put out the memo to all the internal staff’ (e.g., about the availability of transport to appointments) (WG member 5).

WG deliberations enabled community actors to take stock of these changes, describe their impact and identify particular system processes that exacerbated difficulties. This problematization stage in network development enabled them to pool and validate their concerns and translate preoccupations and observations into problem statements to motivate and guide further efforts. As one member stated:

*When a number of groups come together to look at something like access to health and social services, they bring the specificities about local needs*. (WG member 6)

#### 3.2.2 Generating actor interest

In ANT, the second stage in network development involves generating actor interest. The WG adopted three main strategies. The first was reaching out to contacts from the health and care system who could provide a deeper understanding of the problems identified.

*It was a multipronged approach: Let's really try and link with some of the people who understand the workings of the CI(U)SSS, and let's have some conversations with people who understand these access issues*. (WG member 6)

‘Guests’ appeared to welcome the invitation mainly as an opportunity to participate with community actors in problematization: reforms had brought major and rapid changes to working environments, with little opportunity for actors within the system to understand and discuss their impact. These guests were forthcoming with information about the challenges they faced internally, the imperfect rollout of certain plans and their own uncertainties.

A second strategy used by the WG was to participate in public meetings organized by system actors, generating interest in WG issues while also exploring venues available within the system to address their concerns. However, these meetings focused on presenting performance data that did little to clarify how services were being altered or the impact on access. Though a brief question period allowed the expression of concerns, these were not included in the public record for follow‐up. CI(U)SSS representatives repeatedly pointed to two venues available within the public system for users to register complaints and participate in decisions: The Ombudsman's office in each establishment, and the User Committees required by law to exist in health and social service establishments. While public actors stressed that complaints to the Ombudsman were needed to prompt action within organizations, WG members (as well as community residents at the Forum) saw this mechanism as ill‐suited to vulnerable populations most likely to experience problems.

*If you don't have someone to hold your hand through that Ombudsman process, yes it exists, but it's daunting and our people are not up to that*. (WG member 5)

The Ombudsman is designed to receive complaints from individuals, with no opportunity for community groups that hear about people's difficulties and have their trust to accompany them or relay their issues to prompt action. WG members were also somewhat ambivalent about the opportunity for participation provided by User Committees. Reforms had inserted greater distance between User Committees and decision‐making levels in the CI(U)SSS, dislocating User Committees from local territories while extending the range of services each one represented (from long‐term care to ambulatory services). FMGs presented an additional challenge, as they stood outside the purview of user committees, ombudsmen or other communication, participation or accountability mechanisms.

A third strategy used by the WG to generate actor interest was to organize a Community Health Forum, with the objective ‘to provide (neighbourhood) residents, in particular those living in vulnerable situations, an opportunity to obtain information about health and social services in their community and how to access these services’ (WG communiqué). The network mapping in Figure 2 reveals how the Forum enabled the WG to expand network links with other community actors and public‐sector organizations. This was a free public event with kiosks and workshops that offered community residents and public‐sector and community providers a chance to find out about a wide range of services. Presentations by managers, board members and ombudsmen from the CI(U)SSS, as well as FMG physicians, included lengthy Q&A sessions. A second Forum was being planned at the end of the study period. While the WG itself had no funding, some of the groups involved were able to earmark a few thousand dollars to cover expenses (meeting hall, signs, refreshments).

In this second stage of network development, WG members generated interest among system actors to meet with the group and help them understand system changes and processes. They also explored venues within the system where they could pursue further links, and served as an intermediary to create links between system actors and community members.

By coming together in the WG, and assembling their knowledge and experience to validate concerns and identify priority issues, community actors accumulated the social capital needed to interest public system actors in exploring access issues with them and, at the Forum, with community residents. Exchanges enabled a confrontation of perspectives and clarified some of the changes brought about through reforms.

#### 3.2.3 Enrolment and mobilization

Callon and Ferrary^14^ describe the next stages of network maturation in ANT as the assumption of roles within the network that enable mobilization to pursue a consensus mission. The WG represents the creation of a new role, as a community‐based and community‐led venue for exploring concerns around access to health and care services that cut across programme areas (seniors, youth, minorities, etc). The WG enabled community actors to undertake joint deliberation and activities towards a consensus mission. Enrolment of public sector actors remained tentative: several stated that opportunities for exchange with community actors in the WG and at the Forum fed directly into their responsibilities. However, they also expressed discomfort about sharing ‘insider information’ and conflict of interest concerns, which were exacerbated in front‐line professionals by the perceived fragility of their positions within the CI(U)SSS. WG members considered that front‐line workers saw in the WG a valuable advocacy role that they could not play themselves in the system; ‘there's some ambiguity there (about how they see their own role)’ (WG member 5).

Bilodeau et al.^15^ ^(p.169)^ use the term ‘transitional outcomes’ to designate, through modelling based on ANT, events that ‘mark the progression of the action towards its effects’. In the present case, we see the creation, consolidation and expansion of network relations among community actors, and looser network ties achieved with public sector actors (as guests to explore particular issues and speakers at the Forum). The first two stages of network building suggested roles community actors could assume to overcome some access difficulties. For example, once provider guests clarified issues with intake processes, community actors recognized how they could increase accompaniment to help people ‘tell their story’ effectively to assure they were assigned the appropriate priority ranking to obtain services. Other roles included helping people register on the waiting list for a family physician, educating people about available services, and working with public institutions to improve their communication tools (i.e., providing feedback on a new website). WG members contemplated moving into roles within the public system (i.e., User Committees), but remained unsure of their suitability for addressing community concerns. The commitment of public sector decision‐makers to assume new roles with community actors beyond those of ‘guest’ (at WG meetings) and ‘speaker’ (at the Forum) was not evident as a transitional outcome in the present case.

The network development efforts of the WG conferred on community actors the ‘discursive legitimacy’^38^ to speak on behalf of issues as they assembled knowledge of access difficulties faced by community members and understood the system features responsible for these difficulties. During the study period, conditions for coproduction were improved insofar as community actors developed capacities to adapt their services to help people negotiate difficulties. The WG received no sign of system changes to facilitate the integration of these capacities. While system actors appreciated the opportunity to problematize alongside community actors, collaborative mobilization was impeded by conditions created by reforms: insecurity and movement of front‐line personnel, recourse mechanisms ill‐suited to people most likely to have reason to use them and the growing power of physicians over resources without accompanying accountability mechanisms or connection to community actors.

## 4 DISCUSSION

Bovaird defines coproduction as ‘the provision of services through regular, long‐term relationships between professionalized service providers and service users or other members of the community, where all parties make substantial contributions’.^39^ ^(p.847)^ The present case highlights that reforms can produce challenges to coproduction as they disrupt these relationships, and erode the value of existing mechanisms and spaces for sustaining collaborative relationships. The main outcome of WG network‐building efforts lies in the creation of ‘alternate venues’^40^ or ‘organizational infrastructure’^7^ that enables community‐based actors to understand system changes and develop capacities to help people overcome access difficulties in the new context.

We will focus the discussion on three central findings. The first concerns the particular role of community‐driven engagement in creating conditions for coproduction; the second relates to the impact of reforms on the coproduction capacities of public sector actors and the third regards the power dynamics of coproduction that are revealed in this case.

### 4.1 Community‐driven engagement

De Weger et al.^8^ suggests that organizations can create points of connection between communities and local services through forums where citizens and professionals feel comfortable enough to put ideas forward. The results of this study question whether public sector organizations are best placed to perform this role. In contrast to the public meetings of provider organizations, the Community Health Forum provided actionable information (to residents, community groups and public‐sector actors), revealed and explored gaps and, by presenting a vision of health that included a broad range of providers, had the potential to generate solutions outside the public sector to meet needs as well as suggest improvements to public services. Coproduction requires different actors to develop contributions that will be valuable in a given system context. Looking at collaborative strategies in community health, Lasker and Weiss^2^ find that combining the knowledge, skills and resources of a group of diverse participants can lead to ‘breakthroughs in thinking and action’ to strengthen community capacity to solve problems. They recognize the need for ‘“neutral” or “safe” spaces in civic society to support broad‐based collaborative problem solving’^2^ ^(p.41)^ and view problem‐solving processes in civil society as complementary to government's role. ‘Ultimately, it appears that two complementary forms of collaboration are required to strengthen the ability of communities to solve complex problems: one in which the community participates in the work of government and another in which government participates in community‐driven processes in civil society’.^2^ ^(p.41)^ Farmanova et al.,^41^ looking at interfaces between healthcare (medical) and nonhealthcare (or nonmedical/community) services in nine Organization for Economic Cooperation and Development countries conclude that capacity building is needed in both community and healthcare systems, along with greater attention to building and using social capital.

Distinguishing between the two can be complicated by the ‘pre‐history’ of a given system. The Québec context includes venues, such as the ‘tables de concertation’ and user committees, which were designed as opportunities for community participation. Scholars looking at the history of Québec's system note a tendency towards a ‘complémentariste’ view that valued community resources not for their autonomous contributions or insight but for helping to meet Ministry‐determined programme objectives. The ‘tables de concertation’ (in which many WG members participated) were insufficient to address increasing community concerns. Alongside this trend, structural reforms to vertically integrate health and care services into very large organizations divorced health and care services from local communities, weakening the mechanisms for user and community representation in decision‐making.

### 4.2 Impact of reforms on the coproduction capacities of public sector actors

This case study shows that major barriers to the contributions of community actors lie in the fragility and interruption of their links with public sector actors and the lack of clarity about system changes. Québec's system includes front‐line actors with nominal responsibility to support community action and link with community resources—a number participated as guests in the WG. Their hesitancy about the legitimacy of participating points to the disempowerment of these actors through reforms that disrupted their networks within public establishments, as well as their ties to community actors. These findings are in line with Audy and Couturier's^42^ observation that structural and personnel changes in 2015 reforms pose an important threat to the efficacy of networked action. Internal disruption also impedes channels that would allow information drawn from the community to filter into organizational decision‐making. In the context of reforms, the problematization stage of network development appears especially important in understanding barriers to bringing contributions together to effectively use and provide services.

Finally, reforms in Québec give physicians—who remain independent contractors in Canadian systems—greater control over other professionals and public resources, raising new challenges for coproduction that the WG found difficult to address. FMGs are not territory‐based and have no mechanisms for communication with or accountability to communities. Pescheny,^43^ looking at facilitators and barriers to social prescribing in the United Kingdom, concluded that ‘third sector services (community, voluntary, social enterprise) remain underused due to weak links between primary care and the third sector’. The lack of routes into Quebec's FMG model available to community actors represents a break with historical primary care models embedded within communities (though these had progressively weakened before the 2014–2015 reforms).

### 4.3 Power and coproduction

The inadequacy of communication from the public system around changes and access mechanisms during recent reforms in Québec could be attributed to the speed with which reforms were introduced, but contributes to the disempowerment of service users. The consolidation and reduction of service points, movement of front‐line professionals into FMGs and centralized access processes exert a controlling effect on both users and front‐line providers with a net loss of possibilities for both access and recourse. What appears in the case study in Québec is that obfuscating public sector capacity problems behind new unwieldy access processes and poor communication impedes public sector and community capacities to develop solutions to help fill the gaps. From a political philosophy perspective, Badano^44^ recognizes that the emphasis on coproduction in England to ‘harness the renewable energy represented by patients and communities’ and encourage people to ‘feel both free and powerful enough to help themselves and their own communities’ may be a cover for public sector cuts. However, she considers that even if coproduction is pursued with an expressly cost‐control mandate, it might end up being ‘the least possible evil’.^44^ ^(p.20)^ In Quebec, vertical integration and governance changes, coupled with the FMG model of primary care, challenge capacity for action by reducing input from and accountability to local communities.^45^

The present case study reveals that there is considerable work to be done to ensure that community actors understand system changes sufficiently to orient capacity‐building efforts for coproduction. It also reveals that understanding requires the development of power to enlist public sector actors in the problematization effort and supports sociologist Lawrence Benson's^46^ view of interorganizational network development as a means of acquiring power through discursive legitimacy, in this case, to define access problems created through reforms as an essential first step. Coproduction requires a shift in power dynamics so that interdependencies are recognized and acknowledged, and responsibilities redrawn. Through network ties and new venues, community actors may gain the power to induce public bodies to work with them to make gaps clear, negotiate how burdens of care are shifted, clarify new expectations placed on individual and community actors and develop strategies to rebalance responsibilities.

## 5 STUDY LIMITATIONS

The present study explores network development over almost 3 years; however, given the slow pace of progress and the recent nature of reforms, a longer period might reveal more significant role development and greater collaboration between community and public‐sector actors. As well, the study was not designed to capture discussions and actions taken by public sector actors as a result of interaction with the WG network. Research is needed to understand what drives or impedes healthcare providers to establish collaborative relationships with community organizations. The issue of territory as a factor in coproduction also warrants greater attention. Finally, other community‐driven initiatives occurred in the years after reforms, which adopted different approaches to that seen with the WG; comparison might have provided additional insight into community strategies for gaining legitimacy and power in a given reform context.

## 6 CONCLUSION: NETWORK DYNAMICS, POWER AND COPRODUCTION

This study provides a better understanding of the specificities and contribution of community‐driven engagement efforts in developing conditions for coproduction, reveals how network creation enables capacity development and points to venues important in fostering collaborative dynamics between community and system actors. The reform context and attention to history reveal system features that impede coproduction and anticipate challenges associated with moving towards vertically integrated care systems. ANT offers a way to analyse network development towards action through the accumulation of social capital. In particular, this study highlights the significance of network activity in problematization to understand changes brought by reforms as a first step to adapting roles and relationships and (re)creating conditions for coproduction.

## CONFLICTS OF INTEREST

The authors declare no conflicts of interest.

## Data Availability

The data that support the findings of this study are available on request from the corresponding author. The data are not publicly available due to privacy restrictions.
